# Selective modulation of P-glycoprotein-mediated drug resistance

**DOI:** 10.1054/bjoc.2001.2184

**Published:** 2001-12

**Authors:** M Bebawy, M B Morris, B D Roufogalis

**Affiliations:** Faculty of Pharmacy, The University of Sydney, N.S.W, 2006, Australia

**Keywords:** chlorpromazine, colchicine, fluorescein-colchicine, multidrug resistance, P-glycoprotein, vinblastine

## Abstract

Multidrug resistance associated with the overexpression of the multidrug transporter P-glycoprotein is a serious impediment to successful cancer treatment. We found that verapamil reversed resistance of CEM/VLB _100_ cells to vinblastine and fluorescein-colchicine, but not to colchicine. Chlorpromazine reversed resistance to vinblastine but not to fluorescein-colchicine, and it increased resistance to colchicine. Initial influx rates of fluorescein-colchicine were similar in resistant and parental cells, whereas vinblastine uptake was about 10-fold lower in the resistant cells. These results provide indirect evidence that fluorescein-colchicine is transported from the inner leaflet of the membrane and vinblastine from the outer membrane leaflet. Verapamil inhibited fluorescein-colchicine transport in inside-out vesicles made from resistant cells, whilst chlorpromazine was found to activate the transport of fluorescein-colchicine. The chlorpromazine-induced activation of fluorescein-colchicine transport was temperature-dependent and may reflect its interaction with phospholipids localised in the same bilayer leaflet. Conversely, chlorpromazine localisation in this leaflet may be responsible for its allosteric inhibition of vinblastine transport from the opposing membrane leaflet. The proposed relationship between the selectivity of modulation of P-glycoprotein and the membrane localisation of the cytotoxic drug substrates and modulators may have important implications in the rational design of regimes for the circumvention of multidrug resistance clinically. © 2001 Cancer Research Campaign http://www.bjcancer.com


British Journal of Cancer (2001) 85(12), 1998–2003
© 2001 Cancer Research Campaign
doi: 10.1054/ bjoc.2001.2184, available online at http://www.idealibrary.com on IDEAL®

### Selective modulation of P-glycoprotein-mediated drug resistance

**M Bebawy, MB Morris and BD Roufogalis**

Faculty of Pharmacy, The University of Sydney, N.S.W 2006, Australia

**Summary** Multidrug resistance associated with the overexpression of the multidrug transporter P-glycoprotein is a serious impediment to successful cancer treatment. We found that verapamil reversed resistance of CEM/VLB$_{100}$ cells to vinblastine and fluorescein-colchicine, but not to colchicine. Chlorpromazine reversed resistance to vinblastine but not to fluorescein-colchicine, and it increased resistance to colchicine. Initial influx rates of fluorescein-colchicine were similar in resistant and parental cells, whereas vinblastine uptake was about 10-fold lower in the resistant cells. These results provide indirect evidence that fluorescein-colchicine is transported from the inner leaflet of the membrane and vinblastine from the outer membrane leaflet. Verapamil inhibited fluorescein-colchicine transport in inside-out vesicles made from resistant cells, whilst chlorpromazine was found to activate the transport of fluorescein-colchicine. The chlorpromazine-induced activation of fluorescein-colchicine transport was temperature-dependent and may reflect its interaction with phospholipids localised in the same bilayer leaflet. Conversely, chlorpromazine localisation in this leaflet may be responsible for its allosteric inhibition of vinblastine transport from the opposing membrane leaflet. The proposed relationship between the selectivity of modulation of P-glycoprotein and the membrane localisation of the cytotoxic drug substrates and modulators may have important implications in the rational design of regimes for the circumvention of multidrug resistance clinically. © 2001 Cancer Research Campaign http://www.bjcancer.com

**Keywords**: chlorpromazine; colchicine; fluorescein-colchicine; multidrug resistance; P-glycoprotein; vinblastine

Multidrug resistance (MDR) is a serious impediment to the successful treatment of many human cancers. The phenothiazine, chlorpromazine (CPZ), modulates P-glycoprotein (P-gp)-mediated drug transport (Ford, 1996; Syed et al, 1998). We have shown that CPZ and vinblastine (VLB) inhibit each other's transport via P-gp (Syed et al, 1998). However, the P-gp substrate colchicine (COL) fails to inhibit P-gp-mediated CPZ transport (Syed et al, 1996), indicating that the interaction of this drug substrate with P-gp differs from that of VLB.

The reversal of MDR by pharmacological modulators remains poorly understood. Structure–function studies have failed to identify a common molecular requirement for reversal by structurally and functionally dissimilar modulators. As with P-gp substrates, hydrophobicity appears to be a consistent requirement for MDR reversal by modulators, suggesting that their ability to partition into the membrane is important (Zamora et al, 1988).

Many modulators induce significant alterations in plasma membrane properties that could affect the entry of drugs into cells (Wadkins and Houghton, 1993). Membrane structure can also affect P-gp's catalytic cycle, since membrane-active compounds can modulate P-gp-mediated drug transport (Saeki et al, 1991, 1992; Sinicrope et al, 1992; Callaghan et al, 1993; Regev et al, 1999). In addition, membrane-active compounds can affect the binding of drug molecules to P-gp to different extents (Romsicki and Sharom, 1999).

Here we examine the effect of the membrane-active CPZ on both the resistance and P-gp-mediated active transport of fluorescein-colchicine (FC), COL and VLB in the MDR cell line CEM/VLB$_{100}$. CPZ has different effects on both drug resistance and transport with these 3 substrates. We propose that the effect of CPZ on transport of P-gp substrates may depend on the particular leaflet from which each substrate is transported.

*Received 23 January 2001*
*Revised 3 August 2001*
*Accepted 21 September 2001*

*Correspondence to:* BD Roufogalis

### MATERIALS AND METHODS

### Cell culture

The drug-sensitive parental human acute lymphoblastic leukaemia cell line, CCRF-CEM (Foley et al, 1965), and its multidrug-resistant subline, CEM/VBL$_{100}$ (Beck et al, 1979), which was selected for its resistance to 100 ng mL$^{-1}$ VLB, were a kind gift from Dr R Davey (Royal North Shore Hospital, Sydney). Cells were grown as previously described (Bebawy et al, 1999).

### Cell proliferation assay

The cytotoxicity profiles of VLB and COL in the presence and absence of MDR reversal agents were assessed using the CellTiter 96® AQ$_{ueous}$ One Solution Cell Proliferation Assay (Promega, USA). Exponentially growing cells were seeded into sterile 96-well microtitre plates at a density of $5 \times 10^4$ cells/well. Cells were treated with increasing concentrations of VLB or COL (Sigma, USA) in complete RPMI-1640 medium in a final volume of 200 µL in the presence or absence of 10 µM verapamil (VER) (Sigma, USA) or 2 µM CPZ (Sigma, USA). The concentrations of reversal agents chosen for these studies were the highest doses that had no apparent lethal effects on the cells, as assessed from the effects of VER and CPZ alone on cytotoxicity in the cell proliferation assay. The highest non-lethal dose determined for each reversal agent was the same in both the CCRF-CEM and CEM/$VLB_{100}$ cell lines (data not shown).

Control wells contained medium and cells without drugs. Plates were incubated for 72 h at 37˚C in a humidified atmosphere with 5% $CO_2$, after which cells were analysed for viability, 20 µL of $AQ_{ueous}$ One Solution was added to each well, and the cells incubated for a further 4 h at 37˚C in a humidified atmosphere with 5% $CO_2$. Absorbance was read at 490 nm.

Data are expressed as % cell viability ((average absorbance of treated wells/average absorbance of control wells) × 100) versus the test drug concentration. The $IC_{50}$ values were defined as the drug concentration that produced a 50% decrease in cell viability following the 72 h treatment period. From this, the relative resistance towards VLB and COL ($IC_{50}$ of resistant subline/$IC_{50}$ of sensitive parental line) was calculated. Fold reversal in the presence of reversal agents (VER and CPZ) was calculated as the ratio of $IC_{50}$ in the absence of reversal agent/$IC_{50}$ in the presence of reversal agent.

For the trypan blue dye exclusion assay, cells were seeded and incubated as above with increasing concentrations of FC (Molecular Probes, USA). Following the 72 h treatment period, 50 µL trypan blue (0.5%) was added to 50 µL cell suspension and the cells enumerated using a haemocytometer. Data were expressed in terms of % cell viability ((average live cell counts of treated wells/average live cell counts of control wells) × 100) versus FC concentration, in order to determine the $IC_{50}$ values. The relative resistance towards FC for the CEM/$VLB_{100}$ cell line and the fold reversal in the presence of reversal agents were also calculated.

### Measurement of FC transport in inside-out plasma-membrane vesicles

Inside-out plasma-membrane vesicles isolated from CCRF-CEM and CEM/$VLB_{100}$ cells were prepared as previously described (Bebawy et al, 1999). Transport of FC into inside-out vesicles was determined as previously described (Bebawy et al, 1999). Measurements of the transport of 150 nM FC ± CPZ were conducted at 25˚C and 37˚C (0–10 µM). The kinetics of 5 µM verapamil inhibition and 5 µM CPZ activation of FC transport were determined over a range of FC concentrations (0–1 µM). Initial rates of FC transport were calculated from the absolute value of the slope relative to the amount of membrane vesicle protein used in the reaction.

### Measurement of drug uptake into whole cells

Initial rates of drug uptake into whole cells were measured using the method described by Sirotnak et al (1986). Cells were harvested and resuspended in uptake buffer (107 mM NaCl, 10 mM Tris-HCl, pH 7.4, 26.2 mM $NaHCO_3$, 5.3 mM KCl, 1.9 mM $CaCl_2$, 1 mM $MgCl_2$, 7 mM D-glucose) to a density of 2–4 × $10^7$ cells $ml^{-1}$ and used immediately.

Drug influx at 37˚C was initiated upon the addition of either FC or [$^3$H]VLB at final concentrations of 50 µM and 100 nM, respectively. At given times, aliquots of cells were rapidly diluted into 2 ml ice-cold phosphate-buffered saline (Gibco Life Technologies, Aus) to terminate the reaction. Samples were immediately washed twice by centrifugation in the same buffer and the final pellet resuspended in 1 ml solubilising solution (0.5% (v/v) Triton X-100). Radioactivity was quantified using a liquid scintillation counter. Fluorescent samples were analysed using the read application of the FLDM program in the Perkin Elmer LS-50 fluorimeter at excitation and emission wavelengths of 492 nm and 519 nm, respectively.

### Data analysis

$IC_{50}$ values were calculated by fitting the plots of % cell viability versus drug concentration to the inhibitory Sigmoid $E_{max}$ model (Meibohm and Derendorf, 1997) using MicroMath Scientific software (version 2). An unpaired 2-tailed Student's *t*-test was used for statistical analysis between treatment groups. The *a priori* level of significance was set at ($P < 0.05$).

Initial rates of uptake of drug in inside-out vesicles and whole cells were estimated by fitting plots of drug uptake versus time to a polynomial or linear function, respectively, using Kaleidagraph (version 3.0.4). This program uses the Levenberg–Marquardt algorithm for nonlinear regression fits. Plots of initial rate of transport versus concentration of drug were fitted to the Michaelis–Menten equation by non-linear regression using Kaleidagraph (v 3.0.4).

### RESULTS

### Modulation of the cytotoxicity of P-gp substrates

Dose–response cytotoxicity profiles for VLB, COL and FC were established for both the drug-sensitive CCRF-CEM and drug-resistant CEM/$VLB_{100}$ cells ± the reversal agents, VER and CPZ. In the absence of the reversal agents, the resistant CEM/$VLB_{100}$ cells displayed $IC_{50}$ values of 710 ± 75 nM, 430 ± 50 nM and 14.2 µM for VLB, COL and FC, respectively (Figure 1). The $IC_{50}$ values for the CCRF-CEM cells were 1.5 ± 0.1 nM, 8.3 ± 1.2 nM and 1.02 ± 0.01 µM for VLB, COL and FC, respectively (Figure 1). The relative resistance to VLB, COL and FC in the CEM/$VLB_{100}$ cell line was calculated to be 480-fold, 52-fold and 14-fold, respectively, thus confirming that the CEM/$VLB_{100}$ cell line was resistant to VLB, COL and FC relative to the parental line.

10 µM VER reduced the $IC_{50}$ for VLB and FC cytotoxicity of CEM/$VLB_{100}$ cells by 51-fold and 5.6-fold, respectively (Figures 1A, C) ($P < 0.05$). 10 µM VER did not significantly alter the $IC_{50}$ value for COL resistance (Figure 1B). VER had no significant effect on the $IC_{50}$ values for VLB, COL or FC in the parental CCRF-CEM cell line (Figure 1A–C).

2 µM CPZ reduced the $IC_{50}$ for VLB cytotoxicity in CEM/$VLB_{100}$ cells by 8-fold (Figure 1D). The same concentration of CPZ, however, increased the $IC_{50}$ value for COL by 3.6-fold (Figure 1E). CPZ had no significant effect on the cytotoxicity profile of FC (Figure 1F). CPZ had no significant effect on the $IC_{50}$ values for VLB, COL and FC cytotoxicity in the parental CCRF-CEM cell line (Figure 1D–F).

### Effect on fluorescein-colchicine transport in inside-out vesicles by verapamil and chlorpromazine

The mechanism by which VER and CPZ modulated P-gp-mediated transport of 150 nM FC was examined in inside-out vesicles from CEM/$VLB_{100}$ cells. VER inhibited the initial rate of 150 nM FC transport into the CEM/$VLB_{100}$ membrane vesicles in a dose-dependent manner (Figure 2A, inset). Inhibition of FC transport in CEM/$VLB_{100}$ membrane vesicles occurred with a shift in both the $K_m$ and $V_{max}$, indicating that the inhibition was mixed (competitive/non-competitive) (Figure 2A).

*© 2001 Cancer Research Campaign* 

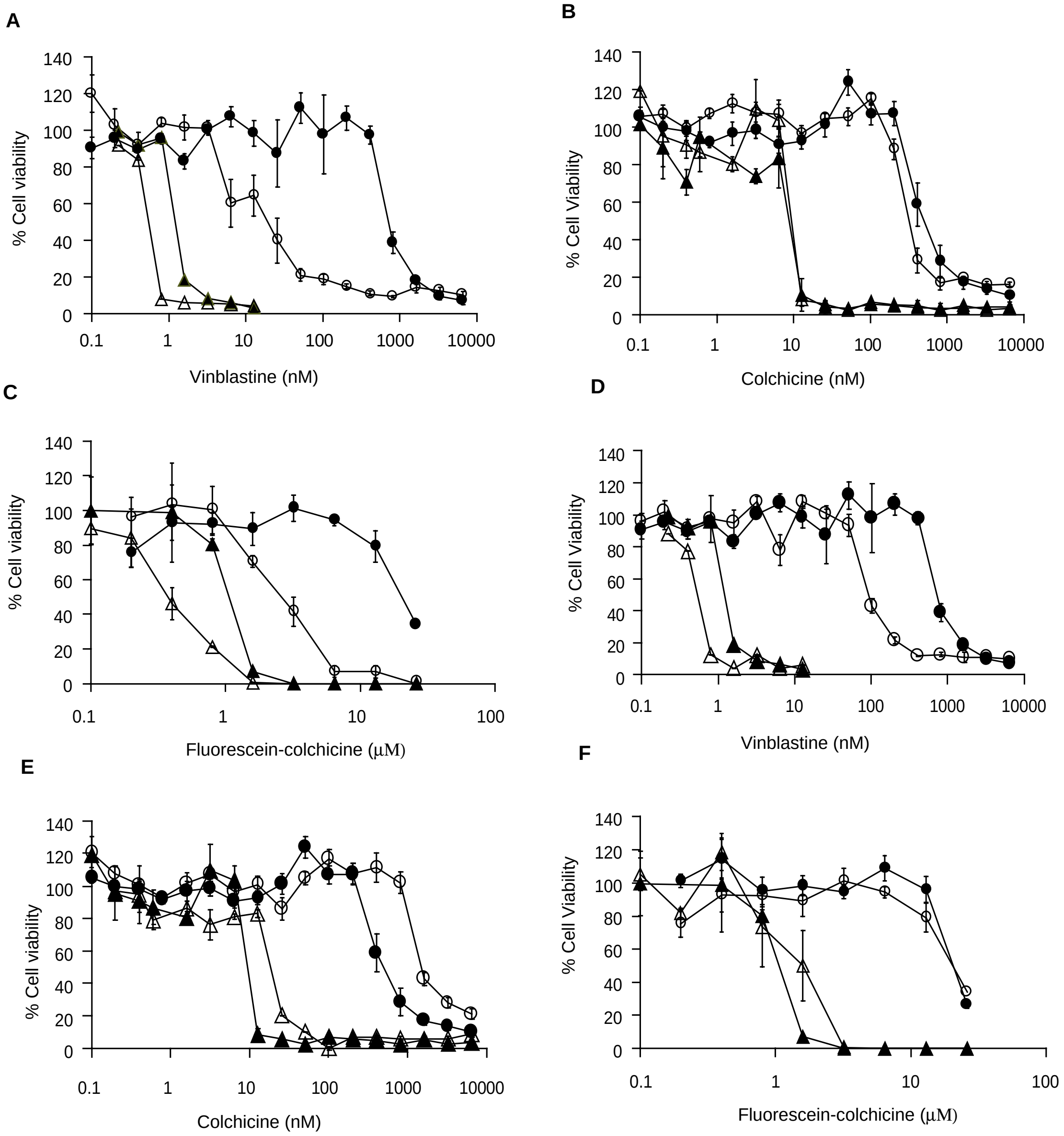

**Figure 1** The effect of VER and CPZ on VLB, COL and FC cytotoxicity in CEM/$VLB_{100}$ cells. 5 × $10^4$ CCRF-CEM (▲, Δ) and CEM/$VLB_{100}$ (○, ●) cells were exposed to increasing concentrations of VLB, COL and FC in the presence (○, Δ) and absence (●, ▲) of 10 μM VER (**A**, **B**, **C**) or 2 μM CPZ (**D**, **E**, **F**) for 72 h. Results are expressed as % cell viability, which is equivalent to the number of viable cells in the presence of VLB, COL or FC divided by the number of viable cells in the absence of these drugs (see Materials and Methods for details). (**A**) VLB ± VER; (**B**) COL ± VER; (**C**) FC ± VER; (**D**) VLB ± CPZ; (**E**) COL ± CPZ; (**F**) FC ± CPZ. The data represent the mean ± SEM

CPZ activated 150 nM FC transport into the CEM/$VLB_{100}$ membrane vesicles in a dose-dependent manner, with up to a 2.6-fold activation at 10 μM CPZ (Figure 2B, inset). The kinetics of activation of FC transport by 5 μM CPZ resulted from a 4-fold decrease in $K_m$. No effect on uptake by CPZ was observed in the absence of ATP (data not shown).

ATP-dependent FC transport in inside-out vesicles was temperature-dependent, and was 4.6-fold greater at 37˚C than at 25˚C (Figure 3A). The effect of CPZ on FC transport was examined at both 25˚C and 37˚C (Figure 3B). At 25˚C, increasing CPZ maximally activated FC transport 8-fold at concentrations ≥ 4 μM. This contrasted with a maximum 2.6-fold activation observed at 37˚C (Figure 3B).

### Initial uptake rates of fluorescein-colchicine and vinblastine in whole cells

No significant difference in the initial rate of uptake of 50 μM FC was observed between the CCRF-CEM and CEM/$VLB_{100}$ cells (Table 1). In contrast, the initial uptake rate for 100 nM [$^3$H]VLB in CCRF-CEM cells was 10 times that of CEM/$VLB_{100}$ cells (Table 1). The reduction in initial rate of VLB uptake in CEM/$VLB_{100}$ cells compared to the CCRF-CEM cells was almost completely reversed in the presence of 10 μM VER (Table 1). The addition of VER had no significant effect on the uptake rate in the CCRF-CEM cell line.

 *© 2001 Cancer Research Campaign*

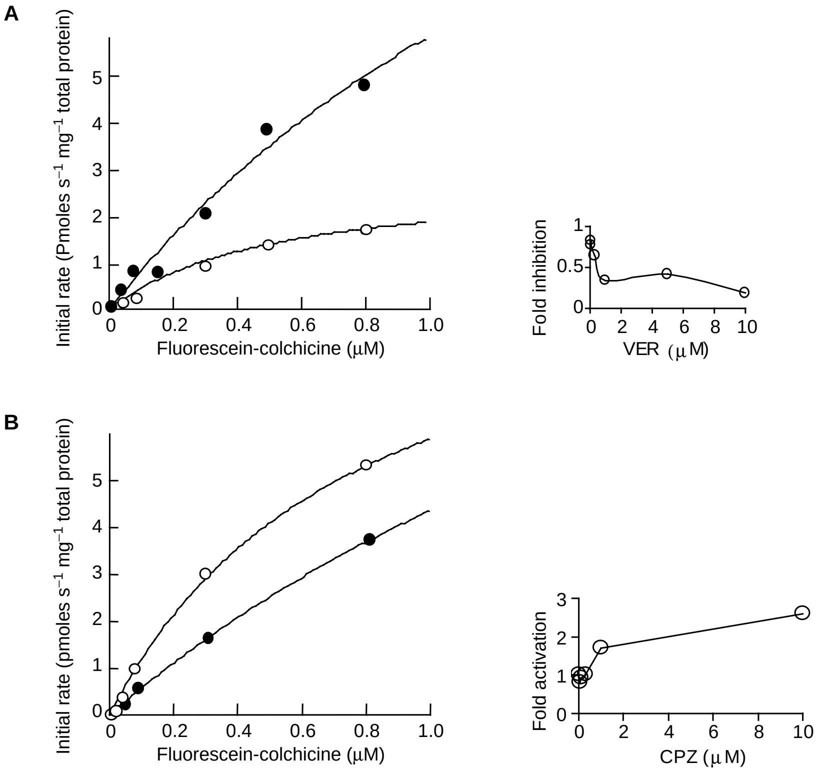

**Figure 2** Effect of (**A**) VER and (**B**) CPZ on FC uptake by inside-out membrane vesicles from CEM/$VLB_{100}$ cells at 37°C. Initial uptake rates were determined at the indicated concentrations of FC in the presence (○) and absence (●) of 5 µM VER or CPZ, as described under Materials and Methods. The solid lines represent the best fit to the data using the Michaelis–Menten equation. The calculated values of the parameters were as follows: (**A**) (○) $V_{max}$ = 18.5 ± 9.1 pmoles $mg^{-1}$ $s^{-1}$, $K_m$ = 2 ± 1.3 µM; (●) $V_{max}$ = 2.9 ± 0.5 pmoles $mg^{-1}$ $s^{-1}$, $K_m$ = 0.5 ± 0.2 µM. (**B**) (●) $V_{max}$ = 16.3 ± 2.6 pmoles $mg^{-1}$ $s^{-1}$, $K_m$ = 2.7 ± 0.5 µM; (○) $V_{max}$ = 10.5 ± 0.8 pmoles $mg^{-1}$ $s^{-1}$, $K_m$ = 0.8 ± 0.1 µM. Inset: Dose-dependent inhibition (**A**) and activation (**B**) of 150 nM FC transport by VER and CPZ, respectively, in CEM/$VLB_{100}$ inside-out membrane vesicles at 37°C. Initial uptake rates were determined at the indicated concentrations of VER or CPZ, as described under Materials and Methods. Data are from one of 3 typical experiments

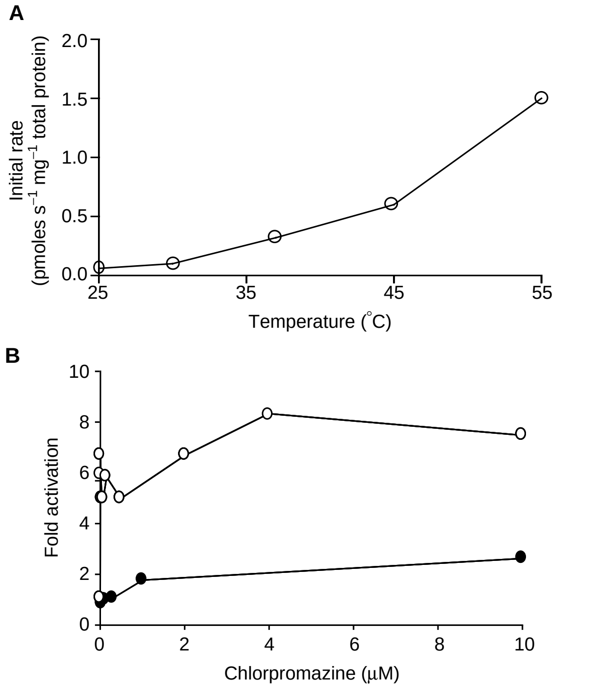

**Figure 3** Effects of temperature on FC initial uptake rate and CPZ activation in inside-out membrane vesicles from CEM / $VLB_{100}$ cells. Initial uptake rates of 150 nM FC were determined (**A**) at the indicated temperatures or (**B**) at increasing concentrations of CPZ at 25°C and 37°C, as described under Materials and Methods. Data are from one of 2–3 typical experiments

**Table 1** Initial uptake rates of 50 µM FC, and 100 nM [$^3$H] vinblastine (VLB) in the presence and absence of 10 µM verapamil (VPL) in CCRF-CEM and drug-resistant CEM/$VLB_{100}$ cells

| Cell line | Condition | Rate ± SEM (pmoles $10^6$ $cells^{-1}$ $s^{-1}$) |
|---|---|---|
| | FC | |
| CCRF-CEM | | 440 ± 12 |
| CEM/$VLB_{100}$ | | 580 ± 60 |
| | [$^3$H]VLB | |
| CCRF-CEM | | 0.004 ± 0.0002 |
| | +VPL | 0.004 ± 0.001 |
| CEM/$VLB_{100}$ | | 0.0004 ± 0.0001[a] |
| | +VPL | 0.003 ± 0.0004[b] |

Initial uptake rates were determined as described in Materials and Methods. [a]$P$ < 0.05 comparing the rate of [$^3$H]VLB uptake in CEM/$VLB_{100}$ to CCRF-CEM control using the unpaired Student's *t*-test ($n$ = 3–4); [b]$P$ < 0.05 comparing the rate of [$^3$H]VLB uptake in the presence of verapamil (VPL) to that in the absence of VPL using the unpaired Student's *t*-test ($n$ = 3–4).

### DISCUSSION

Drug resistance to the P-gp drug substrates, VLB, COL and the fluorescent COL derivative, FC, was assessed in the P-gp-expressing MDR cell line, CEM/$VLB_{100}$, relative to the parental drug-sensitive CCRF-CEM line. We have recently shown FC to be a P-gp drug substrate using the inside-out membrane vesicle model (Bebawy et al, 1999).

© 2001 Cancer Research Campaign 

The multidrug transporter, P-gp, transports various drug substrates differently. This is reflected by differences in the cooperativity of transport of different drugs (Ayesh et al, 1996) and proposed differences in the plasma membrane site at which these substrates interact with the drug transporter (Stein, 1997). Here we provide further evidence for differences in the way substrates are transported and we demonstrate selective modulation of P-gp-mediated MDR by a reversal agent, depending on the type of drug transport.

Whilst VER inhibited (to various degrees) drug resistance to cell cytotoxicity of all the drug substrates examined (Figure 1A–C), CPZ displayed both inhibition and enhancement of drug resistance, depending on the drug substrate. As expected from its inhibitory effects on P-gp-mediated [$^3$H]VLB transport in membrane vesicles (Syed et al, 1998), CPZ reversed VLB resistance in intact CEM/VLB$_{100}$ cells (Figure 1D). The same concentration of CPZ, however, enhanced COL resistance (Figure 1E) and had no effect on FC resistance in the same cells (Figure 1F).

The effects of VER and CPZ on the P-gp-mediated transport of FC in inside-out membrane vesicles from resistant cells were also examined. The results obtained are consistent with resistance to cytotoxicity in the CEM/VLB$_{100}$ cells being, to a large part, dependent on P-gp overexpressed in these cells. VER inhibited the initial rate of FC transport in a dose-dependent manner (Figure 2A, inset), consistent with its reversal of FC resistance in intact CEM/VLB$_{100}$ cells (Figure 1C). The mixed (competitive/noncompetitive) kinetics of inhibition of FC transport by VER (Figure 2A) suggest that the inhibitor acts at a site distinct from the transport site. In contrast, CPZ activated the transport of FC in a dose-dependent manner (Figure 2B and inset). The activation is consistent with the failure of CPZ to reverse COL or FC resistance in whole cells (Figure 1E, F). The activation of transport is associated predominantly with an increased affinity of the transporter for FC (Figure 2B).

To determine if different substrates were localised within different regions of the membrane bilayer, the initial uptake rates of FC and VLB were examined in both the drug-resistant and drug-sensitive cells. From kinetic analysis of the initial rates of uptake of different P-gp drug substrates in whole cells, Stein (1997) proposed that different drug substrates can preferentially interact with P-gp at sites in different halves of the membrane bilayers from which they are subsequently extruded (see Figure 4). Compounds whose initial rate of uptake in resistant cells is reduced relative to their drug-sensitive counterparts are proposed to be extruded from the outer-membrane leaflet, prior to their penetration into the inner-membrane leaflet of the plasma membrane (Figures 4C, D). Compounds having the same initial rate of uptake in sensitive and resistant cells must first accumulate in the inner leaflet before being effluxed by P-gp (Figure 4A, B). Since the initial rate of FC uptake into the drug-resistant CEM/VLB$_{100}$ cells was the same as that observed in the drug-sensitive cells (Table 1), according to Stein's model P-gp-mediated FC extrusion occurs from the inner membrane leaflet. In contrast, the initial rate of VLB uptake was significantly lower in the resistant cells than in the sensitive cells (Table 1), consistent with the proposal that VLB is being extruded from the outer plasma membrane leaflet. VER is able to overcome the reduced rate of [$^3$H]VLB transport in the resistant CEM/VLB$_{100}$ cells (Table 1), confirming the likely involvement of P-gp in the early extrusion process.

Whereas 5 μM CPZ inhibited VLB transport (Syed et al, 1998), it activated FC transport (Figure 2B). The overwhelming majority of CPZ distributes to the inner membrane leaflet (Tenforde et al, 1978). Thus, the leaflet into which CPZ preferentially distributes is the same as that in which we propose FC interacts with the drug transporter (Table 1; Figure 4). Differential effects by CPZ on pump activity have been demonstrated for the $Na^+/K^+$-ATPase: CPZ was found to alter the affinity and the translocation rate of cations interacting with the pump at the internal cationic site, whilst having no effect on cations binding at the external cationic site (Giraud et al, 1981). CPZ has been found to inhibit allosterically [$^3$H]VLB transport in inside-out membrane vesicles (Syed et al, 1998). This is consistent with the fact that CPZ distributes overwhelmingly to the inner membrane leaflet whilst as proposed, VLB interacts with, and is transported by, P-gp at the outer leaflet (Table 1; Figure 4).

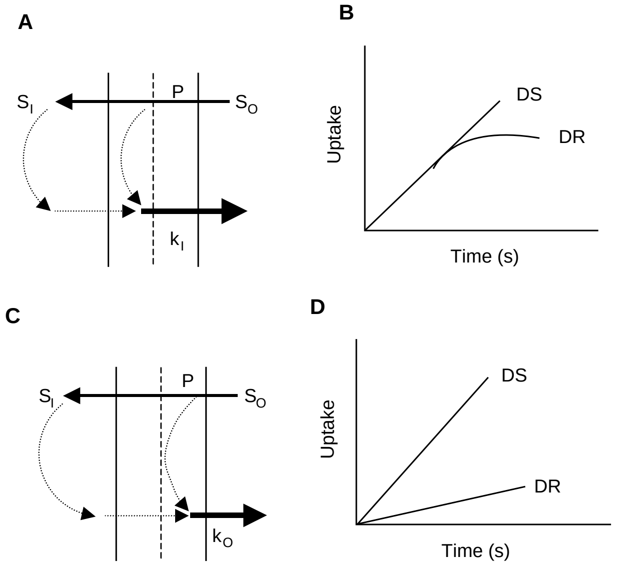

**Figure 4** Schematic diagram representing a model for P-gp-mediated drug extrusion from the inner and outer lipid leaflet of the membrane bilayer (Adapted from Stein, 1997). (**A**) A drug added to the extracellular medium at a concentration $S_o$ and which has a membrane permeability, P, will accumulate within the cell to a concentration $S_I$. The rate constant $k_I$ refers to the rate constant for pumping out the drug from the inner leaflet of the bilayer. At initial times (prior to significant accumulation of the drug within the cell) rates of uptake will be independent of the presence or absence of the pump; i.e., initial rates of uptake will be the same in both drug resistant (DR) and drug sensitive (DS) cell lines (**B**). In (**C**), the pump serves to extrude drug from the outer membrane leaflet with a rate constant $K_O$. As a consequence, at initial times the rate of uptake will be lower in DR cells compared to DS cells (**D**)

CPZ affects membrane properties (Sheetz and Singer, 1976) and at concentrations > 2 μM specifically interacts with the anionic groups of phospholipids, such as phosphatidylserine, located almost exclusively on the inner leaflet of the plasma membrane (Zachowski and Durand, 1988). The activity of P-gp is dependent on a cluster of 53–56 principally inner-leaflet phospholipids, including phosphatidylserine, bound to the transporter (Sharom, 1997). We speculate that the activation by CPZ of P-gp transport of FC results from an interaction between these bound lipids and CPZ. The temperature-dependent effects of CPZ-induced activation on FC transport, in which concentrations ≥ 4 μM CPZ activated FC transport 8 fold at 25°C but only 2.6 fold at 37°C (Figure 3B), may reflect the temperature dependence of changes in the interaction between CPZ and these bound lipids. This may lead to changes in fluidity of the membrane surrounding the P-gp transport site and/or decreased hydrophobic interactions, leading to changes in the conformation of P-gp in the membrane (Sharom, 1997).

 *© 2001 Cancer Research Campaign*

The present study has demonstrated that the modulation of MDR by so-called reversal agents of P-gp is complex, and depends on the site from which a particular substrate is transported by P-gp in relation to the localisation in the membrane of the modulator itself. Dissecting the details of these complex interactions may aid in clinical treatment of MDR with reversal agents selective for different cytotoxic drugs.

### ACKNOWLEDGEMENTS

This research was supported by a Pharmacy Research Trust Scholarship to MB and grants from the Sydney University Cancer Fund.

### REFERENCES

Ayesh S, Shao YM and Stein WD (1996) Co-operative, competitive and non-competitive interactions between modulators of P-glycoprotein. *Biochim Biophys Acta* **1316**: 8–18

Bebawy M, Morris MB and Roufogalis BD (1999) A continuous fluorescence assay for the study of P-glycoprotein-mediated drug efflux using inside-out membrane vesicles. *Anal Biochem* **268**: 270–277

Beck WT, Mueller TJ and Tanzer LR (1979) Altered surface membrane glycoproteins in Vinca alkaloid-resistant human leukemic lymphoblasts. *Cancer Res* **39**: 2070–2076

Callaghan R, Stafford A and Epand RM (1993) Increased accumulation of drugs in a multidrug resistant cell line by alteration of membrane biophysical properties. *Biochim Biophys Acta* **1175**: 277–282

Foley GE, Lazarus H, Farber S, Uzman BG, Boone BA and McCarthy RE (1965) Continous culture of human lymphoblasts from peripheral blood of a child with acute leukaemia. *Cancer* **18**: 522–529

Ford JM (1996) Experimental reversal of P-glycoprotein-mediated multidrug resistance by pharmacological chemosensitisers. *Eur J Cancer* **32A**: 991–1001

Giraud F, Claret M, Bruckdorfer KR and Chailley B (1981) The effects of membrane lipid order and cholesterol on the internal and external cationic sites of the $Na^+$-$K^+$ pump in erythrocytes. *Biochim Biophys Acta* **647**: 249–258

Meibohm B and Derendorf H (1997) Basic concepts of pharmacokinetic /pharmacodynamic (PK/PD) modelling. *Int J Clin Pharmacol Ther* **35**: 401–413

Regev R, Assaraf YG and Eytan GD (1999) Membrane fluidization by ether, other anesthetics, and certain agents abolishes P-glycoprotein ATPase activity and modulates efflux from multidrug-resistant cells. *Eur J Biochem* **259**: 18–24

Romsicki Y and Sharom FJ (1999) The membrane lipid environment modulates drug interactions with the P-glycoprotein multidrug transporter. *Biochemistry* **38**: 6887–6896

Saeki T, Shimabuku AM, Azuma Y, Shibano Y, Komano T and Ueda K (1991) Expression of human P-glycoprotein in yeast cells—effects of membrane component sterols on the activity of P-glycoprotein. *Agri Biol Chem* **55**: 1859–1865

Saeki T, Shimabuku AM, Ueda K and Komano T (1992) Specific drug binding by purified lipid-reconstituted P-glycoprotein: dependence on the lipid composition. *Biochim Biophys Acta* **1107**: 105–110

Sharom FJ (1997) The P-glycoprotein multidrug transporter: interactions with membrane lipids, and their modulation of activity. *Biochem Soc Trans* **25**: 1088–1096

Sheetz MP and Singer SJ (1976) Equilibrium and kinetic effects of drugs on the shapes of human erythrocytes. *J Cell Biol* **70**: 247–251

Sinicrope FA, Dudeja PK, Bissonnette BM, Safa AR and Brasitus TA (1992) Modulation of P-glycoprotein-mediated drug transport by alterations in lipid fluidity of rat liver canalicular membrane vesicles. *J Biol Chem* **267**: 24995–25002

Sirotnak FM, Yang CH, Mines LS, Oribe E and Biedler JL (1986) Markedly altered membrane transport and intracellular binding of vincristine in multidrug-resistant Chinese hamster cells selected for resistance to vinca alkaloids. *J Cell Physiol* **126**: 266–274

Stein WD (1997) Kinetics of the multidrug transporter (P-glycoprotein) and its reversal. *Physiol Rev* **77**: 545–590

Syed SK, Christopherson RI and Roufogalis BD (1996) Chlorpromazine transport in membrane vesicles from multidrug resistant CCRF-CEM cells. *Biochem Mol Biol Int* **39**: 687–696

Syed SK, Christopherson RI and Roufogalis BD (1998) Reversal of vinblastine transport by chlorpromazine in membrane vesicles from multidrug-resistant human CCRF-CEM leukaemia cells. *Brit J Cancer* **78**: 321–327

Tenforde TS, Yee JP and Mel HC (1978) Electrophoretic detection of reversible chlorpromazine. HCl binding at the human erythrocyte surface. *Biochim Biophys Acta* **511**: 152–162

Wadkins RM and Houghton PJ (1993) The role of drug-lipid interactions in the biological activity of modulators of multi-drug resistance. *Biochim Biophys Acta* **1153**: 225–236

Zachowski A and Durand P (1988) Biphasic nature of the binding of cationic amphipaths with artificial and biological membranes. *Biochim Biophys Acta* **937**: 411–416

Zamora JM, Pearce HL and Beck WT (1988) Physical-chemical properties shared by compounds that modulate multidrug resistance in human leukemic cells. *Mol Pharmacol* **33**: 454–462

*© 2001 Cancer Research Campaign*
